# Signaling network prediction by the Ontology Fingerprint enhanced Bayesian network

**DOI:** 10.1186/1752-0509-6-S3-S3

**Published:** 2012-12-17

**Authors:** Tingting Qin, Lam C Tsoi, Kellie J Sims, Xinghua Lu, W Jim Zheng

**Affiliations:** 1 Bioinformatics Graduate Program, Medical University of South Carolina, Charleston, SC 29425, USA; 2 Department of Biochemistry and Molecular Biology, Medical University of South Carolina, Charleston, SC 29425, USA; 3 Department of Biomedical Informatics, University of Pittsburgh, Pittsburgh, PA 15232, USA

## Abstract

**Background:**

Despite large amounts of available genomic and proteomic data, predicting the structure and response of signaling networks is still a significant challenge. While statistical method such as Bayesian network has been explored to meet this challenge, employing existing biological knowledge for network prediction is difficult. The objective of this study is to develop a novel approach that integrates prior biological knowledge in the form of the Ontology Fingerprint to infer cell-type-specific signaling networks via data-driven Bayesian network learning; and to further use the trained model to predict cellular responses.

**Results:**

We applied our novel approach to address the Predictive Signaling Network Modeling challenge of the fourth (2009) Dialog for Reverse Engineering Assessment's and Methods (DREAM4) competition. The challenge results showed that our method accurately captured signal transduction of a network of protein kinases and phosphoproteins in that the predicted protein phosphorylation levels under all experimental conditions were highly correlated (R^2 ^= 0.93) with the observed results. Based on the evaluation of the DREAM4 organizer, our team was ranked as one of the top five best performers in predicting network structure and protein phosphorylation activity under test conditions.

**Conclusions:**

Bayesian network can be used to simulate the propagation of signals in cellular systems. Incorporating the Ontology Fingerprint as prior biological knowledge allows us to efficiently infer concise signaling network structure and to accurately predict cellular responses.

## Background

New proteomics techniques enabled large-scale experiments that monitor phosphorylation states of many proteins under different physiological stimuli and/or pharmacological treatments. Each measurement captures a static picture of how the cellular signaling network responses to the binding of a ligand to its receptor, but the interconnections among many different ligand-activated pathways are complex and dynamic. Thus, it is of biological importance to infer which signaling path is at work in response to a particular ligand and how pathways "cross-talk" to each other in a cell-type-specific manner, and eventually to develop computational models capable of predicting cellular responses under different stimuli.

One of the most common approaches to signaling network modeling is to represent the dynamic system as a set of ordinary differential equations (ODEs) using mass action kinetics, by which the concentration of species over time can be analyzed [1,2]. Additionally, when spatial information is important for such modeling, a system of partial differential equations (PDEs) is considered to be more precise tool to model biochemical processes in both space and time dimension [3-5]. ODEs or PDEs mathematically represent signal transduction by introducing many parameters in the model, which becomes impractical for extremely large networks due to the increasing difficulty in parameter estimation [1]. To approach large-scale signaling network modeling, several data-driven methods have emerged and applied to simulate signal transduction: constraint-based network analysis that allows reconstruction of large systems of biochemical reactions in analyzing genome-scale metabolic networks [6-8]; network component analysis (NCA) which incorporates prior knowledge of network topology to infer signaling pathways [9,10]; partial least squares regression (PLSR) analysis to investigate complicated signaling networks by identifying optimal principle component-based dimensions from a proposed relationship [11-13]. Many of the approaches described above are deterministic models, which are not aimed at accommodating the noise inherent in biologically data [14]. In contrast, Bayesian network analysis is an alternative probabilistic graphical approach to model signaling pathways [15,16]. Bayesian network, which can explicitly handle the uncertainty of unobserved events [14,17], provides a compact graphical representation of the joint probability distributions over all random variables, and has been used for reconstruction of signaling networks [18-24].

To assess the current state of the art network inference methods, Columbia University, the New York Academy of Sciences, and the IBM Computational Biology Center have been organizing the Dialogue for Reverse Engineering Assessments and Method (DREAM), an annual international competition to assess methods that infer network structures and predict cellular response to different combination of stimuli from actual experimental data [25]. Challenge 3 of the 2009 DREAM4 competition (will be referred to as DREAM4 challenge) was titled Predictive Signaling Network Modeling and included two tasks. In the first part, a canonical protein phosphorylation network was provided. This network was constructed by combining pathways from different cell types reported in the current literature. The participants were also provided with a dataset of protein phosphorylation measurements collected from HepG2 hepatocellular carcinoma cells that were treated with various stimuli and inhibitors. The task was to induce a HepG2 cell specific protein phosphorylation pathway out of the canonical network and to build a predictive model of how the cell responds to these stimuli and inhibitors. The second part of the challenge was to use this induced pathway to predict the activities of the phosphoproteins under a new set of perturbations.

The provided canonical pathway consists of a union of the known signaling pathways responding to the following ligands *TNFα, IL1α, IGF-1*, and *TGFα *(see the Methods section for detailed description). The training data consisted of the activities of seven downstream phosphoproteins measured when cells were treated with four cytokine (and control) stimuli in various combinations with four inhibitors at 0, 30 minutes and 3 hours post-stimulation. The test data was generated similarly, but the cells were treated with different combination of stimuli and inhibitors [26-28].

Our approach to this challenge is to employ an enhanced Bayesian network to identify the most plausible HepG2 specific signaling network and to predict the cellular responses to new stimuli. Bayesian network is a directed acyclic graph (DAG) model representing the probabilistic relationships between a set of random variables [16]. Given a signal transduction pathway such as the canonical network of DREAM4 challenge, a Bayesian network can represent the propagation of cellular signal for the biological network in such a way that the state of a downstream phosphoprotein is determined by the states of its upstream kinases, and their relationships can be quantified by conditional probabilities [21]. We could then transform the task of inducing cell-type-specific network as a task to find a subnetwork within the canonical network that explains the observed data as well as possible--a data-driven structure search problem. It is well known that brute force exhaustive search of Bayesian network structure is intractable [29] although different heuristic algorithms exist to address the task. However, solely employing these heuristic algorithms in our setting would fail to utilize a wealth of biological knowledge regarding genes and proteins and their relationships. Ignoring this knowledge may result in a Bayesian network that captures the statistical relationships between the states of phosphoproteins perfectly but does not make any biological sense--a phenomenon referred to as equivalent classes of Bayesian networks in the machine learning field [30,31]. In order to address this problem, we developed a Bayesian network searching algorithm that incorporates prior biological knowledge.

We recently developed the concept of the Ontology Fingerprint from biomedical literature and Gene Ontology (GO) [32]. The Ontology Fingerprint for a gene or a phenotype is a set of GO terms overrepresented in the PubMed abstracts linked to the gene or phenotype, along with these terms' corresponding enrichment p-values [33]. By comparing two genes' Ontology Fingerprints, we can assess their biological relevance quantitatively. Such relevance can be used to assess gene-gene connections for model selection in Bayesian network-based signaling network prediction. Incorporating this information accelerates the network search process and helps to identify biologically sound connections in predicting signaling networks, eventually leading to better models. We thus developed an enhanced Bayesian network method by incorporating the Ontology Fingerprint for model selection. This novel approach was used to predict a signaling network for the DREAM 4 challenge and performed very well, indicating ontology and prior biological knowledge can make a significant contribution to signaling network predictions.

## Methods

**Figure 1:**
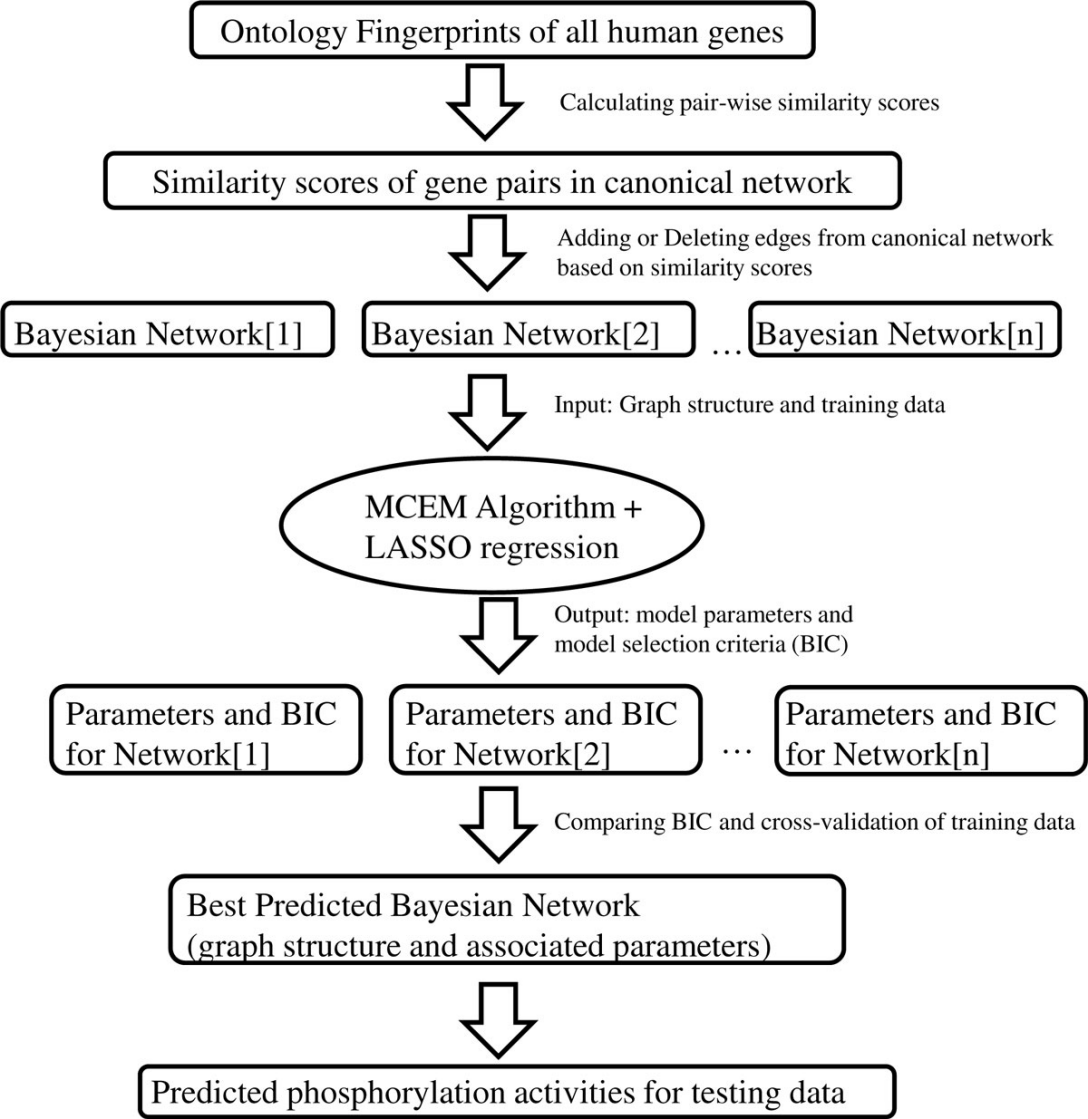
**Schematic representation of the methodology**. The Ontology Fingerprints of the whole human genome were constructed, followed by calculating gene-gene similarity scores using pair-wise comparison of their Ontology Fingerprints. When searching for a cell-type-specific network, the canonical signaling network was repeatedly and stochastically modified by adding or deleting edges based on similarity scores, i.e. the higher the similarity score of a gene pair, the greater possibility of adding the edges connecting the two genes. The candidate networks were trained in parallel using an MCEM (MCMC sampling-based EM) algorithm to infer the states of hidden nodes and estimate network parameters, and LASSO regression was applied in the last round of MCEM. A model selection criteria (BIC) is further calculated for each candidate network. Finally, the best network was selected under the guidance of BIC criteria. The selected network was then applied to predict the phosphorylation activities for the testing data.

**Figure 2:**
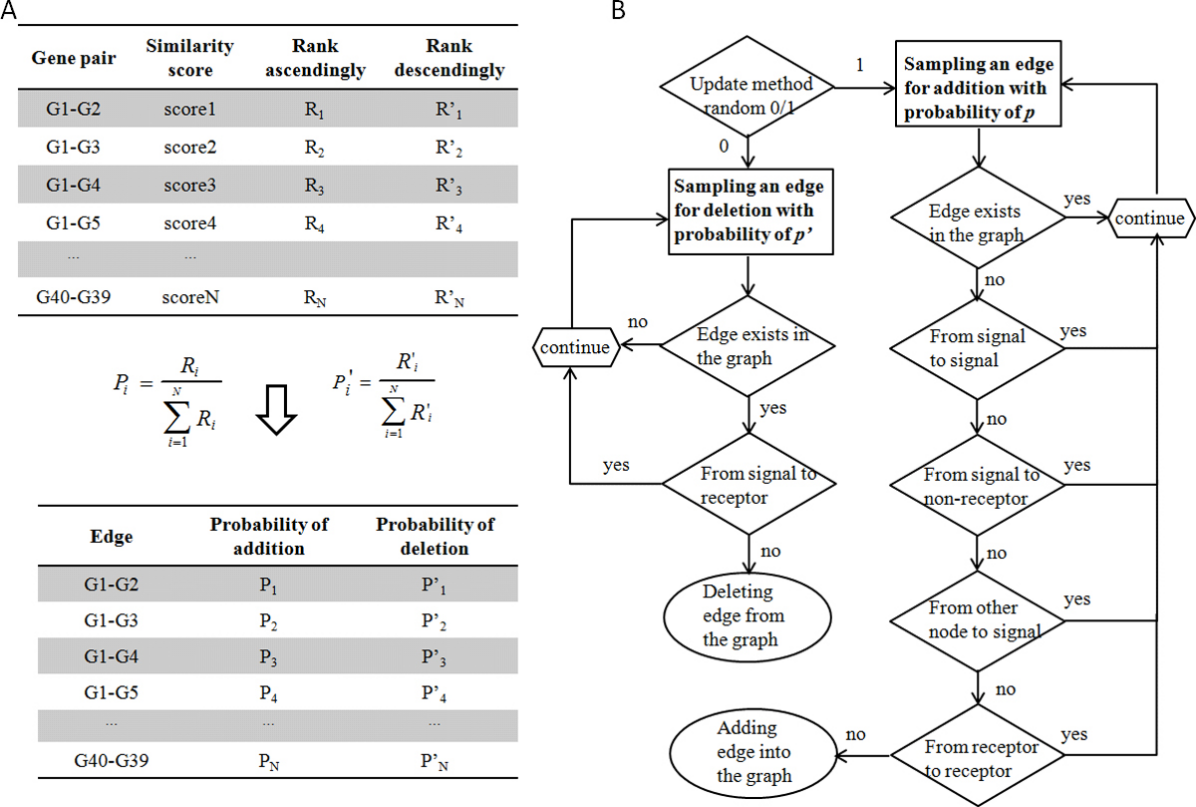
**Heuristic network search algorithm based on the Ontology Fingerprint**. A) The gene-gene similarity scores among the 40 genes of interest were converted into probabilities of adding or deleting edges respectively: i) the similarity scores were ranked in ascending order, and each pair of genes was assigned a corresponding rank R (column "Rank ascendingly"); the probability of adding an edge was obtained by the percentage of its ascending rank out of the total ascending ranks (formula on the left of the arrow); ii) similarly, the probability of deleting an edge was assigned by the percentage of the gene pair's descending rank (column "Rank decendingly") out of the total descending ranks (formula on the right of the arrow). These probabilities ensure that the higher the similarity score of a gene pair, the greater possibility of adding the edge between the two genes; and the lower the similarity score of a gene pair, the more likely the edge between the two genes will be deleted. B) Heuristic rules of adding or deleting edges from the canonical network. A network was updated by either deleting or adding an edge sequentially: i) for deleting edges, an edge was sampled according to its deletion probability (p'); the sampled edge has to exist in the current network and the edges from signals to their corresponding receptors were not allowed to be deleted; ii) for adding edges, an edge was sampled according to its addition probability (p); the sampled edge should not appear in the current network, and the edges between signals, between receptors, between signal and non-receptor, and from other nodes to signal are not allowed to be added.

Combining prior knowledge with experimental data, we adopted a Bayesian network approach to infer the most plausible signaling network from a web of complex networks. Figure 1 outlines the workflow of our method and Figure 2 illustrates the graph-searching algorithm.

### Data

**Figure 3:**
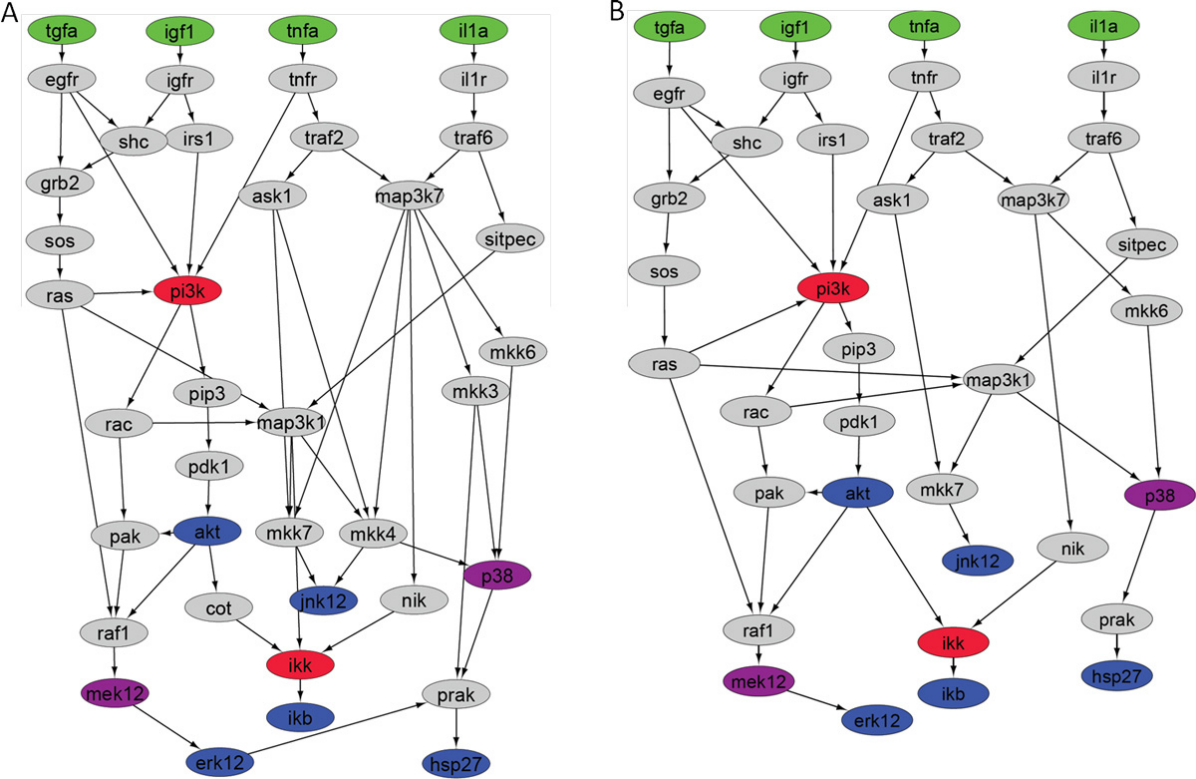
**Full network comparison of the original canonical pathway and the inferred cell-type-specific pathway**. A) Provided by DREAM 4 challenge, the original canonical pathway contains 40 nodes connected by 58 edges: 4 nodes in green represent 4 cytokine receptor which originate signals; 7 nodes in blue or magenta represent observed phosphoprotein with activity measurements; 2 nodes in red represent proteins that are inhibited under some experimental conditions; and 27 hidden nodes in grey have no experimental observation; B) Predicted cell-type-specific pathway activated in HepG2 cell lines, with 37 nodes connected by 47 edges as determined by our algorithm.

The training data were provided by the DREAM4 challenge 3, including phosphorylation measurements for 7 proteins under 25 experimental conditions (combinations of different signal stimuli and kinase inhibition) at 3 time points. We used the provided canonical pathway as the original DAG which contains 40 nodes and 58 edges (Figure 3A). The nodes were classified into four color-coded categories: 1) four ligand receptor nodes (green); 2) seven phosphoprotein nodes whose phosphorylation level were measured as fluorescent signal readings (blue and magenta); 3) two inhibited nodes (red), which were inhibited under some experimental conditions; and 4) hidden nodes (grey). Nodes M*EK12 *and *P38 *are both observed and inhibited nodes under their inhibition condition. In addition, *PI3K *and *IKK *were inhibited in some experiments but their phosphorylation states were not measured.

In order to incorporate independent biological knowledge to learn the network structure, we evaluated the degree of biological relevance between genes by using the gene-gene similarity scores derived from their Ontology Fingerprints; the pairwise similarity scores among the 40 nodes were calculated. The detailed procedures of constructing Ontology Fingerprint were described in [33]. Specifically, we downloaded and processed the June 13th, 2007 version of GO to extract GO terms and their descriptions. The 2007 version of PubMed abstracts in XML format was also downloaded and processed to extract the PubMed ID and the text of each abstract. The links between PubMed abstracts and genes were obtained from the NCBI "pubmed2gene" file. Abstracts that contained GO terms were identified by exact string match. We also labeled the abstracts containing a GO term with all of the term's parent terms. In addition, each abstract was labeled with a GO term only once regardless of how many times the term occurred in the abstract. The ontology fingerprints were derived from 178,687 abstracts linked to at least one human gene. In total, we constructed Ontology Fingerprints for 25,357 human genes using 5,001 ontology terms mapped to the PubMed abstracts that linked to human genes.

### Bayesian network

A Bayesian network was constructed based on the provided canonical signal transduction network, in which nodes are proteins and directed edges represent signaling flows [34]. For the proteins whose phosphorylation signals were measured, we represented their phosphorylation states using Bernulli variables, such that state = 1 (phosphorylated) and state = 0 (unphosphorylated). Under such a setting, the observed fluorescent signals reflecting the phosphorylation level of a protein (the concentration of phosphorylated protein) can be modeled using a Gaussian distribution conditioning on their states (Equation (1)):

(1) $$\begin{array}{c} p\left(v_{i}|s_{i}=0\right)\sim N\left(\mu_{i,0},\sigma_{i,0}\right)\\ p\left(v_{i}|s_{i}=1\right)\sim N\left(\mu_{i,1},\sigma_{i,1}\right) \end{array}$$

Where *v_i _*denotes the activity reading of observed node *i, s_i _*denotes its state; *μ_i_*_,0 _and *μ_i_*_,1 _represent the average activity reading of node *i *at sate 0 and state 1 respectively; *σ*_*i*,0 _and *σ*_*i*,1 _represent the variance of activity readings of node *i *at sate 0 and state 1 respectively. The fluorescent measurements of the seven observed nodes are modeled using a mixture of signals produced by phosphorylated and unphosphorylated proteins.

Under the causal Markov assumption [35-37], we represented the conditional probabilistic relationship between a phosphoprotein and its upstream signaling molecules (kinases) with a logistic function, i.e. given the states of a node *i*'s parents, the probability of the node *i *being at active state (*s_i _*= 1) is independent of its nondescendents' states. This logistic function was defined in Equation (2)

(2) $$p\left(s_{i}=1|pa\left(s_{i}\right)\right)=\frac{1}{1+e^{-\left(\beta_{i,0}+\underset{j\in pa\left(s_{i}\right)}{\sum }\beta_{i,j}s_{j}\right)}}$$

where *pa*(*s_i_*) denotes the set of parent nodes of node *i*, and *j *denotes one of *i*'s parent nodes; *s_j _*represents the state of *j*; *β*_*i*,0 _is the interception and *β*_*i*,*j *_is the logistic regression coefficient between node *i *and its parent node *j*.

### Learning structure of cell-type-specific signaling network

The DREAM 4 challenge requires inferring the cell-type-specific signal network and predicting the cellular response under certain stimulations. We formulated these tasks as learning the structure and parameterization of the Bayesian network and adopted a Bayesian learning approach to determine the structure. Under this framework, the goal is to identify a network structure, a model *M*, which has the maximal posterior probability given data *D *(Equation (3) and (4)):

(3) $$M^{*}=\text{arg}{\text{max}}_{M\in G}p\left(M|D\right)$$

(4) p(M|D)~p(M)p(D|M)

The number of all possible network structures of a Bayesian network *G *(Equation (3)) is super exponential [38,39] with respect to the number of nodes. Thus, exhaustive search of all possible structures is intractable. In this study, we developed a heuristic approach to utilize prior biological knowledge to guide a stochastic search of biologically plausible candidate graphs, equivalent to selecting networks with higher prior *p*(*M*). Based on these candidate networks, we further performed a data-driven search of network structure through parameterization. We identified an optimal cell-type-specific network for HepG2 cells by combining the networks that were preferentially selected based on prior knowledge and that explained the observed data well.

### Searching for biological plausible network using the Ontology Fingerprint

Using the provided canonical network as a starting point, we explored the space of the cell-type-specific networks by stochastically adding and deleting edges. The edge selection was based on the available prior biological knowledge in order to search for network structures that are more biologically sensible. To this end, we employed the Ontology Fingerprint [33] to represent the prior knowledge of proteins of interest. The Ontology Fingerprint of a gene provides the characteristics of the cellular component, molecular function, or biological process captured in the literature with a quantitative measure. By comparing two genes' Ontology Fingerprints using a modified inner product, a similarity score is generated to quantify the gene-gene relationship--the higher the score, the more the two genes are biologically relevant. We used these similarity scores to guide the exploration of model space of possible networks.

We calculated the similarity scores for all pairs of 40 genes in the canonical pathway. The similarity score was used to assess whether an edge should be added or deleted in the canonical network: edges linking two genes with strong biological relevance (i.e. high similarity score) will be added into the network with a higher chance, while edges with weak biological relevance and weak data support will be deleted from the network with a higher chance. Figure 2 shows the heuristic rules of network search. The candidate graphs were then used to infer the parameters by applying the EM algorithm.

### Searching for network structure based on observed data

Given a candidate network produced in the aforementioned space exploration, we further evaluated if the model explains the observed experimental data well by calculating the term *p*(*D*|*M*) in Equation (4). This involves learning the parameters of the network model and considering all possible combination of parameterization of the model to derive the marginal probability *p*(*D*|*M*). In this study, we employed LASSO logistic regression to perform regularized (aka Bayesian) estimation of parameters. We also used the Bayesian information criteria (BIC) [40] as a surrogate of the marginal probability of the network to assess the goodness of fit of the models. In addition, we took advantage of the fact that, when the logistic regression parameter between a target phosphoprotein and one of its parents is set to zero by the Lasso logistic regression, we can effectively delete the edge between these two proteins--searching for network model through parameterization.

### Bayesian learning of network model

The true phosphorylation states of the protein nodes were not observed but indirectly reflected by the fluorescence signals in the training data. Therefore the nodes representing protein phosphorylation states were latent variables. We used an expectation-maximization (EM) algorithm to infer the hidden state of each node and further estimated the parameters of candidate models [41]. The hidden states of the protein nodes were inferred using a Gibbs-sampling-based belief propagation in the EM algorithm, i.e. Monte Carlo EM algorithm (MCEM) [42]. In the E step, the state of a node was inferred based on the states of its Markov blanket nodes using a Gibbs sampling algorithm, and all the nodes' states were updated following the belief propagation algorithm. In the M step the parameters associated with edges were estimated based on the sampled states of the nodes. The Markov blanket of node *X *is a set of nodes consisting of *X'*s parents, children, and other parents of *X*'s children nodes. Given the states of the nodes within *X*'s Markov blanket, the *X*'s state is independent of the states of nodes outside the Markov blanket. We derived the full conditional probability of a hidden node (Equation (5.1) - (5.3)):

(5.1) $$p\left(s_{i}=0|MB\left(s_{i}\right)\right)=\frac{p\left(s_{i}=0|pa\left(s_{i}\right)\right)p\left(ch\left(s_{i}\right)|pa\left(ch\left(s_{i}\right)\right),s_{i}=0\right)}{D_{h}}$$

(5.2) $$p\left(s_{i}=1|MB\left(s_{i}\right)\right)=\frac{p\left(s_{i}=1|pa\left(s_{i}\right)\right)p\left(ch\left(s_{i}\right)|pa\left(ch\left(s_{i}\right)\right),s_{i}=1\right)}{D_{h}}$$

(5.3) $$\begin{array}{ccc} D_{h}= & p\left(s_{i}=0|pa\left(s_{i}\right)\right)p\left(ch\left(s_{i}\right)|pa\left(ch\left(s_{i}\right)\right),s_{i}=0\right) & \\ & +p\left(s_{i}=1|pa\left(s_{i}\right)\right)p\left(ch\left(s_{i}\right)|pa\left(ch\left(s_{i}\right)\right),s_{i}=1\right) \end{array}$$

Similarly, the full conditional probability of the observed node was described in Equation (6.1) - (6.3), where the probability of each node's state conditioned on the states of its parents (*p*(*s_i_*|*pa*(*s_i_*))) can be determined using Equation (2):

(6.1) $$\begin{array}{ccc} p\left(s_{i}=0|MB\left(s_{i}\right)\right) & =\frac{p\left(v_{i}|s_{i}=0\right)p\left(s_{i}=0|pa\left(s_{i}\right)\right)p\left(ch\left(s_{i}\right)|pa\left(ch\left(s_{i}\right)\right),s_{i}=0\right)}{D_{o}} & \\ & =\frac{N\left(\mu_{i,0},\sigma_{i,0}\right)p\left(s_{i}=0|pa\left(s_{i}\right)\right)p\left(ch\left(s_{i}\right)|pa\left(ch\left(s_{i}\right)\right),s_{i}=0\right)}{D_{o}} \end{array}$$

(6.2) $$\begin{array}{ccc} p\left(s_{i}=1|MB\left(s_{i}\right)\right) & =\frac{p\left(v_{i}|s_{i}=1\right)p\left(s_{i}=1|pa\left(s_{i}\right)\right)p\left(ch\left(s_{i}\right)|pa\left(ch\left(s_{i}\right)\right),s_{i}=1\right)}{D_{o}} & \\ & =\frac{N\left(\mu_{i,1},\sigma_{i,1}\right)p\left(s_{i}=1|pa\left(s_{i}\right)\right)p\left(ch\left(s_{i}\right)|pa\left(ch\left(s_{i}\right)\right),s_{i}=1\right)}{D_{o}} \end{array}$$

(6.3) $$\begin{array}{ccc} D_{o}= & N\left(\mu_{i,0},\sigma_{i,0}\right)p\left(s_{i}=0|pa\left(s_{i}\right)\right)p\left(ch\left(s_{i}\right)|pa\left(ch\left(s_{i}\right)\right),s_{i}=0\right) & \\ & +N\left(\mu_{i,1},\sigma_{i,1}\right)p\left(s_{i}=1|pa\left(s_{i}\right)\right)p\left(ch\left(s_{i}\right)|pa\left(ch\left(s_{i}\right)\right),s_{i}=1\right) \end{array}$$

Logistic regression was then used in the M-step to estimate the parameters of the generalized linear model. In order to reduce the search space, LASSO regression implemented in the LARS package from R [43] was applied in the final round of the EM algorithm to determine whether to perform regularization. This would set certain parameters to zero between a parent-child protein pair in the candidate network [44,45] while retaining the edges that were sufficient to model the observed data. Lasso regression could thus reduce the number of edges in networks that have weak or duplicated effect on signaling cascade.

### Prediction of test data

To predict the fluorescent signals of 7 phosphoproteins in response to cytokine stimuli under 40 testing conditions, the phosphorylation states of these proteins were sampled using the aforementioned EM algorithms (E step only) and the belief propagation algorithm. The fluorescent signals were then simulated by mixture of the signals of proteins in both phosphorylated and unphosphorylated states defined in Equation (1). We generated 50 samples of the activation state for each protein node according to its posterior probability and each sample predicted the strength of fluorescent signal of the monitored proteins from the learned normal distribution conditioned on sampled states. The final prediction was then produced by averaging the predicted measurements of the observed nodes across all samples.

## Results

The task of learning cell-type-specific network is equivalent to determining which subset of vertices and edges from the canonical network should be retained for that cell type. We addressed the task of learning network structure through combining prior knowledge and experimental data in the following steps: 1) stochastically exploring candidate network structures based on prior knowledge; 2) training candidate Bayesian network using experimental data, which further modifies network structure through parameterization, i.e., setting the parameters associated with certain edges to the values that would be equivalent to deleting these edges; and 3) selecting the network model that best simulates the experimental results. A Bayesian network can also readily simulate the propagation of a signal in the system using a belief propagation algorithm [29], which can predict the system's response to cellular stimuli.

The novelty of our approach is to update the network by leveraging prior biological knowledge captured in the Ontology Fingerprints [33] in order to efficiently search for better network structure. The similarity of the Ontology Fingerprints of a pair of genes captures their biological relevance, e.g. whether they participate in a common biology process within a common biological setting such as the same cell type. Therefore, two genes with similar Ontology Fingerprints are more likely to cooperatively work in a common biological environment than those that are not. This information could be used as prior knowledge to preferentially retain or reject the edges in the canonical network in a principled manner.

### Learning cell-type-specific signaling network

**Figure 4:**
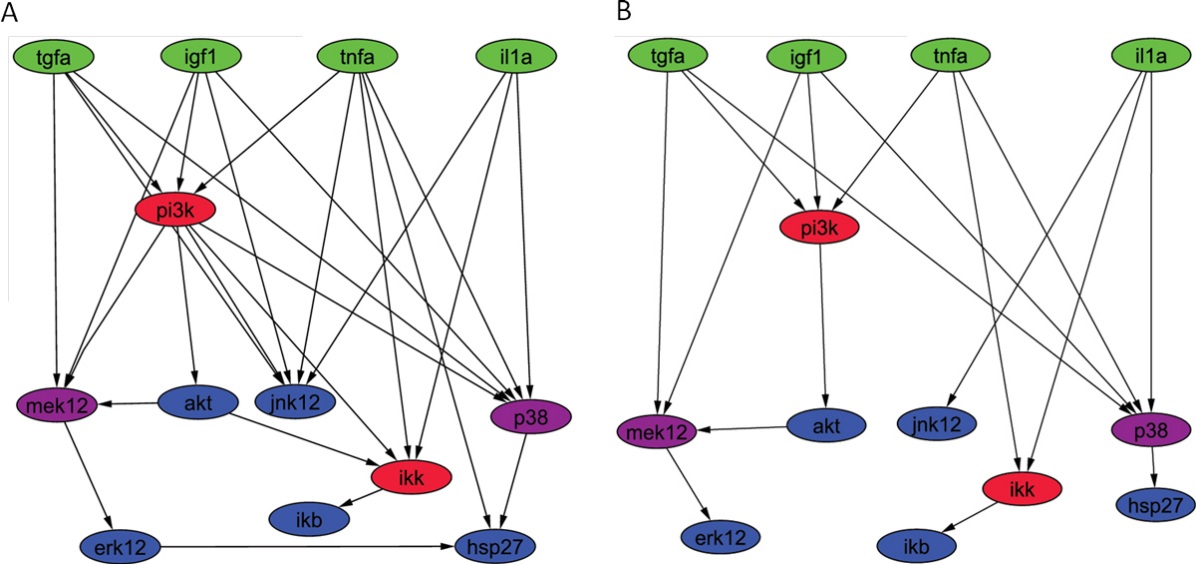
**Comparison of the collapsed original canonical and the inferred cell-type-specific pathways**. A) Collapsed canonical network provided by DREAM4 challenge where all hidden nodes and corresponding edges are removed; B) Collapsed network predicted by our Ontology-Fingerprint-based graph search algorithm.

Using the provided experimental data, we trained our Bayesian network-learning algorithm to infer a HepG2 cell specific network. Figure 3A shows the provided canonical network and the final predicted network is shown in Figure 3B. DREAM4 competition only required to report a collapsed graph, i.e. all hidden nodes removed, and only the paths among the observed phosphoproteins (colored nodes) shown. Figure 4 shows the comparison between the collapsed canonical network and the network learned by our algorithm. The figure shows that the learned graph is simpler than the canonical graph: it contains 17 edges instead of 27 in the canonical network. Notably, the number of each receptor's edges was reduced to three, resulting in a narrower transduction path for each receptor. An intermediary node (*PI3K*) lost all outgoing signals except one, and two terminal nodes (*ERK1/2 *and *HSP2/7*) lost their connecting edge. Another intermediary node (*JNK1/2*) lost its incoming signals from three of the four signal nodes (*TGFa, IGF1 *and *TNFa*).

The predicted network represents a biologically plausible signaling pathway specific to HepG2 cells, partially due to the novel graph search algorithm based on the Ontology Fingerprints. For instance, the connections between *IKK *and *IKB *tended to be kept during graph updating due to the relatively high similarity of their Ontology Fingerprints, with the similarity score ranking above the 80^th ^percentile. In contrast, the connection between *ERK1/2 *and *HSP2/7 *was deleted with a high probability since their similarity score lies on the 30^th ^percentile. Overall, the model updating process based on the novel graph search algorithm seamlessly included prior biological knowledge embedded in the literature and GO. Based on the training data of HepG2 cell, employing LASSO regression [46] in learning Bayesian network parameters further identifies main paths specifically transducing the signal in this cell type, resulting in a sparse network.

Our results also indicate that Bayesian network is particularly suitable for modeling cellular signal transduction in that principled statistical inference algorithms, e.g., the belief propagation algorithm, enabled us to represent hidden variables (nodes without observations) in the graph and to infer detailed signal transduction in the pathway. In contrast, other modeling approaches reported at the DREAM4 conference, e.g., methods based biochemical systems theory [47], usually ignore all hidden variables to reduce the complexity of network modeling and parameter estimation at the cost of missing intermediate information. The full network predicted by our approach consists of 37 nodes connected by totally 47 edges, and each edge is associated with a parameter that quantifies the relationship of the signal propagated from the parent node to its child node (Figure 3B). In this network, twenty-four nodes are hidden but our inference algorithm correctly inferred their states and relationships between the nodes in the network. For instance, the directed edge from *RAS *to *RAF*, both of which are hidden nodes, was assigned with a positive coefficient (*β *= 53.12), indicating that *RAS *plays a strong activation role for *RAF1*. This infer-ence agrees with previous findings [48-50] that *RAF1 *is a critical *RAS *effector target, and its activation is a consequence of *RAS *activation and the formation of *RAS*-GTP-*RAF1 *complex. The positive connection between another pair of hidden nodes, from *RAC *to *PAK *(*β *= 16.49), is also consistent with the fact that *PAK *is the downstream effector of *RAC *[51,52]. Zimmerman and Moelling [53] suggested that *AKT*-mediated phosphorylation of *RAF1 *leads to the inhibition of the Raf-MEK-ERK cascade and the modulation of the cellular response [54,55]. Indeed, our algorithm correctly captured this relationship, which assigned a negative coefficient (*β *= -1.17E-12) to the edge from *AKT *to *RAF1 *in our predicted network. While the low coefficient may reflect the fact that the inference used the measurement performed on two distal nodes--AKT and ERK, the negative value is indeed consistent with the known inhibitory effect. These evidences demonstrate that our approach can utilize the limited observed data to infer the signal transduction of the full network, even though the state of certain nodes are not observed.

### Predicting cellular responses to stimuli

**Figure 5:**
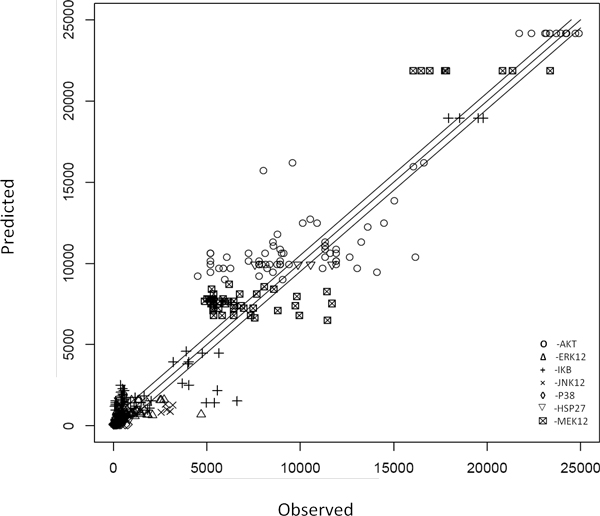
**Comparison of predicted and observed phosphoprotein activity of 7 proteins of interest across different experimental conditions**. We used trained Bayesian network to predict the phosphorylation activity of the 7 proteins of interest under all experimental conditions in the training data set. The "predicted" results were compared with the provided observations and a correlation analysis shows significant correlation (R^2 ^= 0.93).

**Figure 6:**
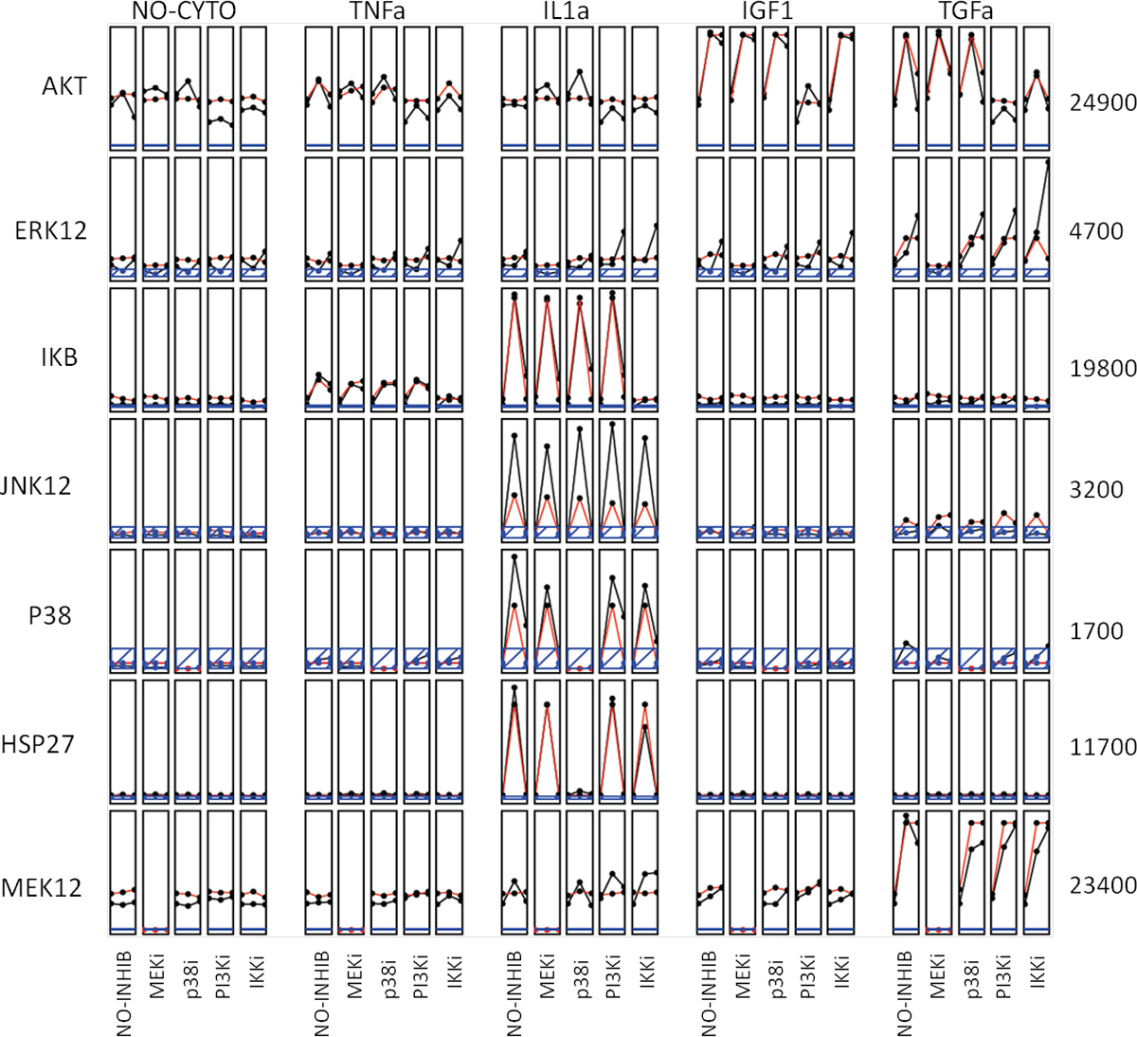
**Phosphorylation activity plots of 7 proteins of interest under the treatments of 5 different stimuli**. We used the trained Bayesian network to predict the phosphorylation level of 7 phosphoproteins under all conditions and compared with the observed data in time-course plots. Within each box, the phosphorylation activity were predicted or observed at 3 time point (0 min, 30 min, and 3 hours post stimulus are plotted, in which the observed data are shown in black and predicted data are shown as red. The blue lines appearing in some boxes indicate that the activity measurement lies within the noise error of the detector (the reading is less than 300).

Using the final graph and the associated parameters learned from the Bayesian network approach, we performed simulation studies to predict cellular responses to a set of provided stimuli and compared the "predicted" results with the observed training data. The comparison showed a very significant correlation (R^2 ^= 0.93). Figure 5 shows the scatter plot between the predicted versus the observed levels for the phosphoprotein activity of all 7 proteins under all conditions. Figure 6 compares the fitting of the data under different conditions for each of the 7 proteins. The black curves denote the observed phosphoprotein activity levels, while the red curves represent the corresponding predicted values. The blue-line within each box indicates the detection threshold of the detector (~ 300). Overall, the predictions are highly consistent with the observed data, indicating that our model is able to capture the signal transduction in HepG2 cells with a sparse network.

Using the predicted HepG2 specific network and the learned parameters, we then predicted the phosphoprotein activity levels of the 7 proteins under the test conditions given by the DREAM 4 Challenge. The predicated phosphoprotein activities were evaluated against experimental measurement by the organizers of DREAM4 challenge using two criteria: first, the accuracy evaluated by a prediction cost function (sum of squared errors over all the predictions); second, network parsimony. Our group (Team 451) ranked within the top five (#4 or #5 depending on different DREAM4 ranking methods) among all submissions for this challenge (http://wiki.c2b2.columbia.edu/dream/results/DREAM4/?c=3_1). This outcome demonstrates that incorporating prior biological knowledge in the form of the Ontology Fingerprint with statistical algorithms for graph searching and parameter estimation can significantly outperform many other approaches for signaling network inference. Our results also demonstrate a novel way to integrate ontological data and literature in learning signaling network construction, as well as the feasibility of applying ontology as biological information in other challenging data-mining problems.

## Discussion

A signaling network is a complex and dynamic system that governs biological activities and coordinates cellular functions [56,57]. Defects in signal transduction are responsible for diseases such as cancer, autoimmunity, and diabetes [58]. By understanding signaling networks, mechanisms of diseases can be investigated more specifically, and the disease could be targeted and treated more efficiently. Moreover, different cell types often activate different parts of signaling networks, resulting in different responses to the same perturbation. In this study, we addressed the DREAM4 challenge of predicting signaling networks using two innovative approaches: 1) by incorporating prior knowledge in the form of the Ontology Fingerprint, we efficiently and preferentially search biologically plausible models, and 2) by using LASSO regression, we unified the Bayesian network parameter learning and structure learning in a data-driven manner. These improvements are principled from a statistical learning point of view and sensible from a biological point of view.

Participants of the DREAM4 challenge developed various computational approaches to model the signaling network and predict their cellular responses to different stimuli. Dynamic mathematical modeling implemented in a system of differential equations is one of the mainstream approaches [59,60]. The method represents signal transduction as detailed and biochemically realistic mathematical equations with the need to estimate many free parameters. However, the parameter estimation becomes extremely challenge as the number of species in the network increases [1]. To circumvent this pitfall, one of the participant teams using this approach omitted all hidden nodes, i.e. species not subjected to experimental manipulation or measurement. Such simplification resulted in missing information of network topology and intermediate signal transduction. An alternative approach is to depict the signaling pathway as a logical model and utilize a two-state discrete (Boolean) logic to approximate the signal propagation in the network. However, the Boolean model is a deterministic approach not rigorous enough to capture real biological events. Furthermore, this model also involved node compression process to remove non-identifiable elements [26].

By contrast, Bayesian network analysis represents an effective mean to encode both the prior knowledge of network topology and the probabilistic dependency in signaling networks [19,61]. This approach has the advantage of being able to handle hidden nodes in a principled manner and to model mixed information of both the noisy continuous measurements and the discrete regulatory logic by modeling these nodes as latent variables and infer novel signaling paths from observed data. Such advantage is particularly useful in real world application where experimental measurements are expansive and limited to certain selected proteins. The utility of these data can be maximized by using latent variables to infer novel signaling paths that contain proteins not been measured. However, the application of Bayesian network in real world modeling is limited due to the super exponential space one has to search in order to identify the optimal model [62]. Compared with other approaches applied in the DREAM4 challenge, our approach has several significant advantages: 1) it is able to predict the discrete state of proteins in a probabilistic manner under different stimuli, without the requirement of node compression; 2) the incorporation of prior biological knowledge embedded in the Ontology Fingerprint accelerates the search for optimal network topology, in other words, it increases the probability of obtaining an optimal network within limited learning time; 3) the Ontology Fingerprint enhanced network search process makes the inferred network more biologically sensible; 4) the LASSO model regularization method efficiently assist the search for a sparse network.

Our algorithm was further improved by embedding biological information from the Ontology Fingerprint into the learning stage of the Bayesian network modeling. This was accomplished through the introduction of prior distributions for the variables. The seamless integration of prior knowledge into the Bayesian network framework allowed us to construct a cell-type specific signal transduction pathway and to use the pathway to predict novel perturbation outcomes in the DREAM4 competition. The Ontology Fingerprint derived from PubMed literature and biomedical ontology serve as a comprehensive characterization of genes. Compared to current gene annotation, the Ontology Fingerprints were generated by a largely unsupervised method, thus do not need well-annotated corpus which is difficult to assemble. In addition, the enrichment p-value associated with each ontology term in an Ontology Fingerprint can be used as a quantitative measure of biological relevance between genes--a feature that is lacking in current gene annotations. This comprehensive and quantitative characterization of genes works well as prior knowledge in our graph searching strategy. In contrast, commonly used graph searching algorithms, such as genetic algorithms, only rely on a randomized exhaustive search that is not able to utilize useful prior information. This limitation not only makes these algorithms inefficient in searching the plausible model space but also potentially lead to networks that are biologically irrelevant.

To assess the contribution of the Ontology Fingerprints to Bayesian network learning algorithm, we compared the likelihoods of Bayesian networks iteratively updated with or without the guidance of prior knowledge derived from the Ontology Fingerprints. Starting with the canonical network, we iteratively updated network structure until a fixed number of networks were obtained. The converged likelihood of each network was obtained by Monte Carlo EM algorithm (MCEM) [42]. The likelihoods from Ontology Fingerprint-guided network update were significantly higher than those without the guide (Wilcoxon signed-rank test, p-value = 3.4 × 10^-2^). In addition, we investigated the performance of Ontology Fingerprint enhanced Bayesian network in eliminating biologically irrelevant relationships from the network. We randomly added edges with similarity scores of zero into the canonical network, and considered the new network as a noisy network. Starting with this noisy network, we performed the same comparison as described above, and the resulting likelihoods from Ontology Fingerprint-guided network update were also significantly higher than the update process without prior knowledge (Wilcoxon signed-rank test, p-value = 1.5 × 10^-3^). Furthermore, the network update with prior knowledge successfully identified and eliminated noisy edges quickly at the first several iterations. These results demonstrated that integrating the Ontology Fingerprint as prior knowledge can speed up the convergence of likelihood, resulting in the increased efficiency of both identifying optimal network structure and retaining biological meaningful connections in the final network.

In addition to prior knowledge, our approach also employed the LASSO technique [46] to select a plausible model in a data driven manner. LASSO is one of the regularization algorithms originally proposed for linear regression models, and has become a popular model shrinkage and selection method. The LASSO method combines shrinkage and model selection by automatically setting certain regression coefficients to zero [63]. This approach effectively deleted certain candidate edges between signaling molecules, and helped to remove redundant variables to obtain a concise model in the final step.

## Conclusion

By incorporating prior biological knowledge, utilizing advanced statistical method for parameter estimation and modeling unobserved nodes as latent variables, we developed a novel approach to infer active signaling networks from experimental data and a canonical network. Our results demonstrated that these improvements allow us to predict signaling network structure and responses that match closely to those identified by experimental approaches.

## Competing interests

The authors declare that they have no competing interests.

## Authors' contributions

WJZ initiated the idea of incorporating the Ontology Fingerprint for network prediction and guided the development of the Ontology Fingerprints. TQ and LCT worked on the method development and signaling network prediction. KJS advised the biological knowledge about the signaling pathway. XL advised the Bayesian network development. TQ and LCT drafted and WJZ and XL finalized the manuscript. WJZ supervised the overall development of the project. All authors have read and approved the manuscript.
